# Mutation of the *MYH3* gene causes recessive cleft palate in Limousine cattle

**DOI:** 10.1186/s12711-022-00762-2

**Published:** 2022-10-29

**Authors:** Anne Vaiman, Sébastien Fritz, Christian Beauvallet, Mekki Boussaha, Cécile Grohs, Nathalie Daniel-Carlier, Anne Relun, Didier Boichard, Jean-Luc Vilotte, Amandine Duchesne

**Affiliations:** 1 Université Paris-Saclay, INRAE, AgroParisTech, GABI, 78350 Jouy-en-Josas, France; 2 INRAE, Oniris, BIOEPAR, 44300 Nantes, France; 3 Clinic for Ruminants, Oniris, 44300 Nantes, France

## Abstract

**Background:**

The palate is a structure separating the oral and nasal cavities and its integrity is essential for feeding and breathing. The total or partial opening of the palate is called a cleft palate and is a common malformation in mammals with environmental or hereditary aetiologies. Generally, it compromises life expectancy in the absence of surgical repair. A new form of non-syndromic cleft palate arose recently in Limousine cattle, with animals referred to the French National Observatory of Bovine Abnormalities since 2012. Since the number of affected animals has increased steadily, this study was undertaken to identify the cause of this disease.

**Results:**

Based on pedigree analysis, occurrence of cleft palate in Limousine cattle was concordant with an autosomal recessive mode of inheritance. Genotyping of 16 affected animals and homozygosity mapping led to the identification of a single disease-associated haplotype on *Bos taurus* chromosome (BTA)19. The genome of two affected animals was sequenced, and their sequences were compared to the ARS-UCD1.2 reference genome to identify variants. The likely causal variants were compared to the variant database of the 1000 bull genome project and two fully linked mutations in exon 24 of the *MYH3* (*myosin heavy chain*) gene were detected: a 1-bp non-synonymous substitution (BTA19:g.29609623A>G) and a 11-bp frameshift deletion (BTA19:g.29609605-29609615del). These two mutations were specific to the Limousine breed, with an estimated allele frequency of 2.4% and are predicted to be deleterious. The frameshift leads to a premature termination codon. Accordingly, mRNA and protein analyses in muscles from wild-type and affected animals revealed a decrease in *MYH3* expression in affected animals, probably due to mRNA decay, as well as an absence of the MYH3 protein in these animals. MYH3 is mostly expressed in muscles, including craniofacial muscles, during embryogenesis, and its absence may impair palate formation.

**Conclusions:**

We describe a new form of hereditary cleft palate in Limousine cattle. We identified two fully linked and deleterious mutations, ultimately leading to the loss-of-function of the MYH3 protein. The mutations were included on the Illumina EuroG10k v8 and EuroGMD v1 SNP chips and are used to set up a reliable eradication strategy in the French Limousine breed.

**Supplementary Information:**

The online version contains supplementary material available at 10.1186/s12711-022-00762-2.

## Background

Palatogenesis refers to the formation of the palate during embryogenesis. In this tightly regulated process, two palatal mesenchymal buds that originate from medial nasal and maxillary prominences grow vertically along the developing tongue and, as the tongue descends in the oral cavity with the first muscular movements, they grow horizontally and they fuse together with a remodelling epithelial-mesenchymal transition (for a review, see [1, 2]). Each of these steps (vertical growth, elevation, horizontal growth, and fusion) is critical for palatogenesis and any failure may result in a cleft palate, i.e. an incomplete or absent fusion of the palatal buds.

Cleft palate (CP) is one of the most common congenital craniofacial abnormalities in humans, with 1/700 births affected [3]. It can be isolated (“pure” CP), associated with cleft lips, or syndromic, such as in the Pierre Robin syndrome in humans. The Veau classification, which is one of the three main ways used to classify CP [4], is based on its morphological description. It comprises four classes: Veau I, for the clefts of the soft (muscular) palate, Veau II, for the clefts of the soft and hard (bony) palates, up to the incisive foramen, Veau III, for the clefts of the soft and hard palates extending unilaterally through the alveolus (gums), and Veau IV, for the clefts of the soft and hard palates that extend bilaterally through the alveolus. Two supplementary classes were added for minor forms of CP: bifid uvula and submucous CP [5]. Since a cleft palate leads to abnormal communication between the nasal and oral cavities, it causes feeding and breathing defects in animals, as well as auditory and speech defects in humans [6]. To overcome these disabling consequences, surgery is still the only option in humans.

CP is also common in other mammals. In mice, almost 200 mutations in different genes, including microRNA, have been associated with this phenotype, which has helped to shed light on the molecular basis of palatogenesis [7, 8]. Several forms of pure or syndromic CP have been described in domestic animals [9, 10]. Online Mendelian Inheritance in Animals (www.omia.org) includes more than 25 phenes in animals among which cleft palate is present (see Additional file 1: Table S1). Although it is rare in cattle, CP has been described since a long time in several breeds, and is more frequently syndromic [10] than isolated. Syndromic CP can have an environmental origin, such as the ingestion of teratogenic plants during a critical period of embryogenesis. Ingestion of alkaloid-containing lupines (e.g. *Lupinus sericeus* and *Lupinus caudatus*) between days 40 and 70 of gestation gives rise to the “crooked calf disease”, which is characterized by either arthrogryposis, spinal deformation (torticollis or scoliosis), or a combination of these lesions, possibly associated with CP of variable severity in several beef breeds [11, 12]. A congenital syndrome including fully penetrant CP, arthrogryposis and kyphoscoliosis was described in Hereford calves, and it was suspected to arise from the toxic effects of exposure to selenium (e.g. due to the presence of selenium-accumulating plants such as *Astragalus pectinatus* and *Astragalus racemosus* in the pasture), only, or in combination with manganese deficiency [13].

A genetic origin was proven for a subset of syndromic CP cases in cattle. In Charolais cattle, cases of arthrogryposis-palatoschisis syndrome (SAP) were first reported in the 1960’s in France, with CP in approximately two thirds of the affected cases [14, 15]. An autosomal recessive mode of inheritance with incomplete penetrance was proposed [16, 17], with probable modifier genes in Charolais cattle, which could explain the low penetrance in pure-bred Charolais compared to crossbred animals [18]. Heterozygous carrier females may have a reproductive advantage compared to wild-type females [19]. Although the causative mutation has not been found, the neurogenic origin of the disease has been shown, with an abnormal neuromuscular differentiation leading to secondary skeletal abnormalities such as CP [15, 20]. In France, the frequency of SAP has significantly decreased in the last decades.

A hereditary origin was also proposed for the “crooked calf disease” in a US herd of Hereford cattle [12]. In Belgian Blue cattle, a mutation was found in the *phosphatidylinositol glycan anchor biosynthesis class H* (*PIGH*) gene, which is associated with autosomal recessive arthrogryposis and CP (with an incomplete penetrance) [21]. In Red dairy cattle, bovine arthrogryposis multiplex congenita is another form of autosomal recessive arthrogryposis, which is associated with CP and skeletal deformations. This disease has been shown to arise from a mutation in the *cholinergic receptor nicotinic beta 1 subunit* (*CHRNB1*) gene [22].

The presence of CP is always highly deleterious for calves, since communication between the oral and nasal cavities leads to aspiration of milk or water and may result in either starvation or aspiration bronchopneumonia [12], but also to dehydration. As a result, the life expectancy of calves suffering from CP is very low, especially because if surgery is an option, it is usually not performed, for financial reasons. Thus, when a novel form of CP arises, investigating its aetiology is important. Although environmental forms exist, it may be possible to decrease the frequency of specific mutations leading to this condition, and thus to decrease the overall number of CP-affected calves.

In France, the emergence of CP-affected animals was described in the Limousine breed from 2012 onwards, with cases reported to the French National Observatory of Bovine Abnormalities (ONAB, www.onab.fr). Most of the affected animals displayed an isolated CP (class Veau II) and only a minority of them were associated with other defects. As the number of cases increased in the progeny of a few sires, we hypothesized that this disease was hereditary and we undertook a genetic analysis in order to identify the causal mutation and to significantly reduce the risk of CP in the Limousine breed.

## Methods

### Bovine sample selection, tissue collection and DNA extraction

**Fig. 1:**
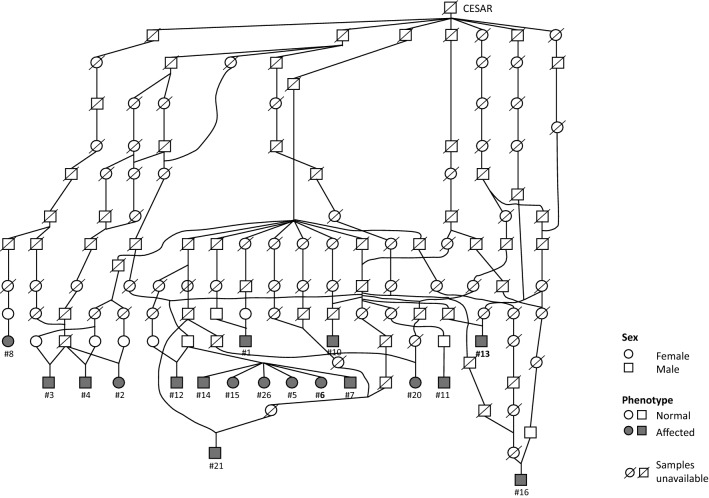
Pedigree of 18 Limousine calves affected by cleft palate. Pedigree data were available for 18 affected animals. Females are represented by circles, males by squares. Filled symbols represent affected animals, their ID number as referred in Additional file 2: Table S2 are written below with a hashtag (#). Two affected animals written in bold (#6 and #13) had their whole genome sequenced. Symbols with a diagonal line show animals for which no DNA sample was available

Twenty-six Limousine cattle presenting with CP (associated or not with other clinical signs) have been recorded by the ONAB since 2012 (see Additional file 2: Table S2). Veterinarians clinically examined 22 of these 26 individuals. Pedigree data were available for 18 of them (Fig. 1) but not for the other eight animals, which were bred by natural mating.

Blood samples were collected from 25 affected animals, 20 available parents of affected animals and three relatives (half-sibs of the affected animals or of the parents). DNA was extracted using the Wizard® Genomic DNA Purification Kit (Promega, France). Extraocular muscle tissue from two CP-affected calves and three healthy age-matched calves (considered as wild-type (WT) animals) was sampled post-mortem and frozen at − 80 °C for RNA/protein extraction.

### Genotyping and mapping

To map the location of this defect, a genome-wide homozygosity scan was carried out using genotyping data from 16 identified affected animals and from 7831 control Limousine animals using different Illumina SNP chips including the BovineSNP50 Beadchip v1 and v2 [23] and the Eurogenomics EuroG10K [24] (Illumina Inc., San Diego, CA). To recover all BovineSNP50 marker information, an imputation step was processed with the FImpute software [25] and using the standard French genomic selection pipeline, as previously described [26, 27]. The resulting genotypes were subsequently analysed using an in-house implementation of the HOMAP (homozygosity mapping) tool [28].

### Whole-genome sequencing and analysis

Whole-genome sequencing was performed at the Get-PlaGe platform (http://genomique.genotoul.fr/) on a HiSeq 3000 Illumina sequencer producing 150-bp-long paired end reads, according to the manufacturer's protocol. The genomic DNA of two affected animals [#6 and #13, (see Additional file 2: Table S2)] was sequenced. All sequences were aligned to the ARS-UCD1.2 reference genome sequence with the Burrows–Wheeler aligner (BWA-v0.6.1-r104) [29]. Then, small genomic variants were identified using the GATK-HaplotypeCaller software [30] as previously described [31, 32]. These variants were compared to those present in the genomic variant database available in the run8 of the 1000 bull genomes project for 4318 animals [31], and to 394 animals from several cattle breeds that have been sequenced by our laboratory [32–34]. All the variants were annotated with the Ensembl variant effect predictor (VEP) pipeline v106 [35] based on the Ensembl version 106 transcript set and using the dbSNP build 143. The effect of the amino acid changes was predicted using the SIFT [36, 37] and PROVEAN [38] prediction software.

### PCR amplification and Sanger sequencing

To validate the identified variants after whole-genome sequencing, exon 24 of the *MYH3* (*myosin heavy chain 3*) gene was amplified using 200 ng of DNA, on a Master Thermal Cycler (Eppendorf) with the GoTaq G2 Flexi DNA polymerase kit (Promega) and with 35 amplification cycles: 94 °C/20 s; 60 °C/30 s; 72 °C/30 s. The primers used were MYH3ex24F 5′-GGCATCCTTCACTCACATCAT-3′ and MYH3ex24R 5′-AGCTGGAGGCTAAGATCAAGG-3′. Then, Sanger sequencing was performed according to standard protocols (Eurofins MWG).

### Estimation of the allelic frequency of the mutations in *MYH3*

The two likely causal mutations were included in duplicate in the Illumina EuroG10k v8 and EuroGMD v1 SNP chips that are used routinely for genomic selection in France. In total, 834,792 animals from 14 French commercial breeds were genotyped for these two mutations.

### RNA extraction, RT-PCR and RT-qPCR

Total RNA was extracted from extraocular muscle tissue from two WT and two CP animals using the Qiazol reagent (Qiagen) and the RNeasy mini kit (Qiagen) with DNase I treatment. Then, 1 µg RNA was reverse-transcribed using an RT-vilo kit (Invitrogen), according to the manufacturer's instructions. To sequence transcripts, cDNAs were amplified by PCR on a Master Thermal Cycler (Eppendorf) with the GoTaq G2 Flexi DNA polymerase kit (Promega) and with 35 amplification cycles: 94 °C/30 s; 62 °C/1 min; 72 °C/1 min. We used primers spanning exons 21 to 27 of the *MYH3* gene: MYH3ex21.1 5′-GGGGCAGTTCATTGACAGTAA-3′ and MYH3ex27.2 5′-TGCTCATCTTCCACTTTGCTT-3′. Following PCR, products were electrophoresed on a 1–2% agarose gel, with ExactLadder DNA PreMix 2 Log (Ozyme) and amplified cDNA fragments of the *MYH3* gene were sequenced (Eurofins MWG).

RT-qPCR was performed in triplicates on extraocular muscle cDNA samples from two WT and two CP animals using the Power SYBR Green PCR Master Mix (Applied Biosystems) on Quantstudio 12k flex Real Time PCR system (Applied Biosystems), with standard PCR conditions. The primers used were MYH3ex23F: 5′-TGAACTGGCCAAGTCAGAGG-3′ and MYH3ex23R: 5′-ACTTGTAGCTGCAGGTCGTT-3′. The *ribosomal protein L13a* (*RPL13A*) and *succinate dehydrogenase complex flavoprotein subunit A* (*SDHA*) genes were used for normalization, with the following primers: RPL13A-1F: 5′-CTGTGAGGGCATCAACATTTC-3′ and RPL13A-1R: 5′-GGAGGGGTTGGTGTTCATC-3′; SDHA-2F: 5′-ACCCGCCGTTTTCAGTTCAC-3′ and SDHA-2R: 5′-AAACTCATGGTCCACAACTGG-3′. These genes were used as stable reference for healthy muscles from several species [39–41]. To calculate the relative gene expression values, we used the geometric average of *RPL13A* and *SDHA* Cts as a reference, as described previously [42].

### Western blot analysis

The protocol for western blot analysis was adapted from [43]. Muscle tissue from two WT and two CP animals was crushed in liquid nitrogen and cytoplasmic proteins were extracted with a 20 mM Tris–HCl pH 7.4, 25 mM KCl, 5 mM EDTA, 5 mM EGTA, 1 mM DTT and 1% Triton X-100 buffer containing the Protease Inhibitors cocktail (Roche). Samples were centrifuged (10 min at 4 °C, 1500×*g*), and the supernatants were retained. Myosin isoforms were extracted from the supernatants with a solution containing 30% glycerol, 25 mM DTT, 2.3% SDS, 62.5 mM Tris–HCl pH 6.8 and traces of bromophenol blue. Proteins were heated 10 min at 60 °C, centrifuged (5 min at room temperature, 8000 rpm), and the supernatants were collected. The 2-D Quant kit (GE Healthcare) was used to determine protein concentration. Fifty µg of protein were mixed with 2× Laemmli sample buffer, loaded on 10% SDS-PAGE and electrophoresed at 80 V for 20 min and 150 V for 50 min, alongside 10 μL of Precision Plus Protein Dual Color Standards (Biorad). After migration, the gels were either Coomassie Blue-stained, or the resolved proteins were transferred onto a nitrocellulose membrane using Trans-Blot Turbo (Bio-Rad) at 2.5 A and 25 V maximum for 10 min. The membrane-bound proteins were then stained with a 5× Red Ponceau solution and destained in TBS-Tween 20 and 0.1% (TBST) for 15 min. The membrane was blocked with 5% non-fat lyophilized milk in TBST for 1 h at room temperature, and primary hybridization was carried out overnight at 4 °C with rabbit anti-MYH3 antibody (GTX32147 batch 821903796, Genetex) (1/1000 in TBST 5% non-fat lyophilized milk). After three washes in TBST at room temperature, the membrane was incubated during 1 h with an anti-rabbit horseradish peroxidase-conjugated secondary antibody (1/10000). Signals were detected using the ECL prime enhanced chemiluminescence system (GE Healthcare) and a ChemiDoc Touch Imager system (Bio-Rad).

## Results

### A new hereditary form of cleft palate in the Limousine breed

**Fig. 2:**
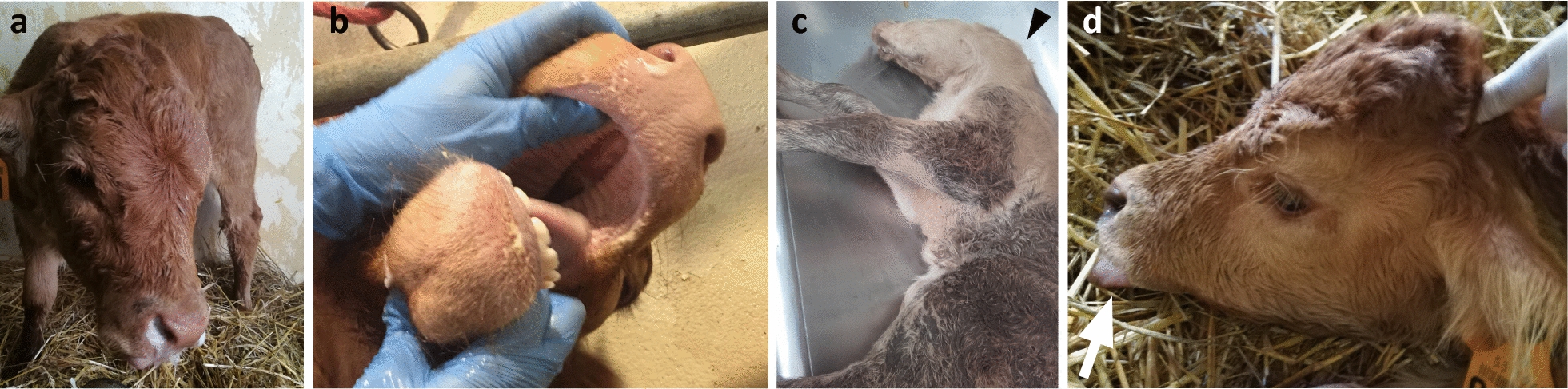
Clinical characterization of Limousine calves affected by cleft palate. **a** Affected calves display bilateral discharge after suckling. **b** Cleft palate affects both the hard and soft palates and can be classified as a Veau II form of CP. **c** Some calves displayed a bent/twisted neck as shown with the black arrowhead. **d** Two calves exhibited macroglossia/tongue protrusion, as shown with the white arrow

Twenty-six Limousine calves presenting with CP were recorded by the ONAB since 2012 (see Additional file 2: Table S2). Bilateral nasal discharge after suckling was observed in the calves shortly after birth (Fig. 2a). Life expectancy was generally short; most animals were euthanized in the first 2 weeks of life due to their difficulty to feed. However, five calves reached 1 month of age or more. In these five affected animals, chronic lower airway infection was often reported, due to dysphagia. CP was classified as Veau II, as it always affected both the soft and hard palates (Fig. 2b). CP was non-syndromic for approximately two thirds of the cases. In the remaining one third, other clinical signs were associated with CP, such as a stunted growth (6/26), bent/twisted neck (3/26; Fig. 2c), vertebral column deformations, for example kyphosis or scoliosis (3/26), macroglossia (2/26; Fig. 2d), or joint deformations (1/26).

Pedigree data were obtained from 18 of the 26 affected animals (Fig. 1). The parents of the affected animals displayed a normal phenotype. Pedigree analysis was consistent with autosomal recessive inheritance. One bull (CESAR FR1925501C08), born in 1967, was identified as the most probable common ancestor for all these cases.

### Two variants in the *MYH3* gene are associated with cleft palate

**Fig. 3:**
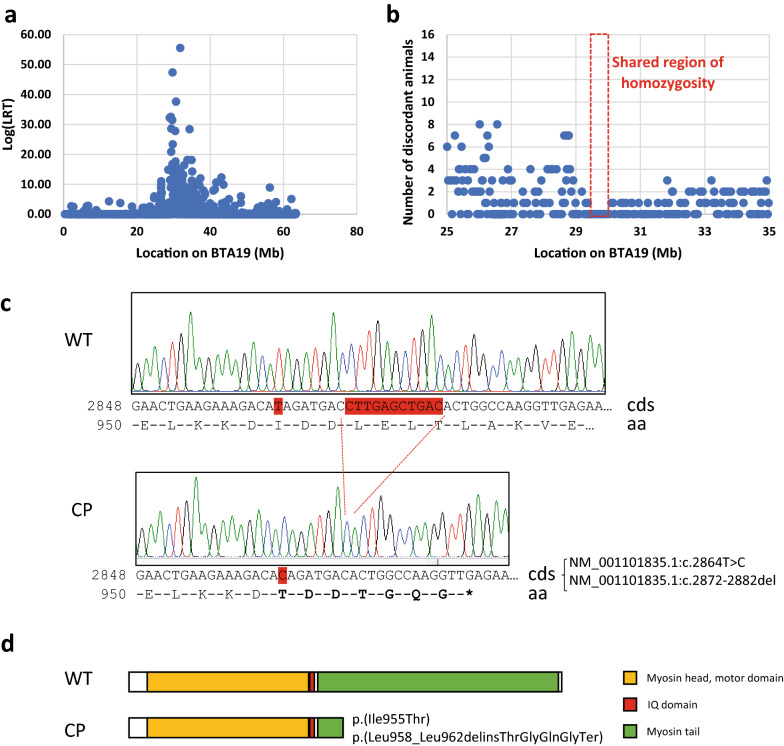
Identification of the cleft palate mutations in the *MYH3* gene. **a** Results of homozygosity mapping on BTA19 on the ARS-UCD1.2 reference genome. **b** Number of discordant animals among the 16 affected animals. The region of homozygosity is defined by the homozygous haplotype on BTA19 shared by all affected animals, from bp 29,396,440 to 30,048,251. **c** Electropherograms of a wild-type and a cleft palate-affected animal after PCR amplification with primers MYH3ex24F and MYH3ex24R. Since *MYH3* is located on the reverse strand, mutations are referred to respectively as T/C for the substitution and CTTGAGCTGACA/A for the deletion to match with the transcription sense. *Cds* coding sequence, *aa* amino-acid, *WT* wild-type, *CP* cleft palate. **d** Scheme presenting the MYH3 protein domains and the predicted consequences of the mutations on the MYH3 protein. *IQ* calmodulin binding domain, *WT* wild-type, *CP* cleft palate

Consistent with the autosomal recessive mode of inheritance, homozygosity mapping was performed on 16 CP-affected animals. The most significant haplotype shared by all affected animals was on *Bos taurus* chromosome (BTA)19 (Fig. 3a) and (see Additional file 3: Fig. S1) on the ARS-UCD1.2 reference genome [44]. The number of discordant animals among the 16 affected animals is indicated in Fig. 3b. The shared runs of homozygosity (ROH) are defined by the homozygous haplotype shared by all affected individuals, from bp 29,396,440 to 30,048,251 (ARS-UCD1.2 reference genome). The maximum likelihood-ratio test (LRT) value (depending on the distribution of genotypes in this group and on allelic frequencies in the control population) observed in this region of homozygosity reached 47. The SNP at bp 31,838,276 is the most significant one because the allele in affected animals is very rare in the overall population, but it is not in this region of homozygosity because three affected animals are discordant at this position.

High-throughput sequencing was performed on two affected animals (cases # 6 and #13 in Fig. 1), which were relatively distant in the pedigree. The mean genome coverages were 23.6X and 19.7X, respectively for cases #6 and #13. Their sequences were compared to the ARS-UCD1.2 reference genome to identify variants. Then, the likely causal variants were compared to the variant database of the 1000 bull genome project (run8), and to 394 animals from several cattle breeds sequenced by our laboratory. Variant analysis was focused on the region of interest identified by homozygosity mapping on BTA19 (from 29,396,440 to 30,048,251 Mb). The detected variants (SNPs and small indels) were filtered in several steps as described in [45]. Briefly, due to the recessive mode of inheritance of the disease, the affected animals had to be homozygous for the causative variant. Second, since the condition described here is assumed to be specific of the Limousine breed, variants were discarded if they were present in other breeds in the data from the 1000 bull genome project and from our laboratory. Finally, due to the deleterious effect of the mutation, variants were filtered according to their predicted effects on the transcript and/or the protein.

Two variants remained: a 1-bp non-synonymous substitution (BTA19:g.29609623A>G) and a 11-bp frameshift deletion (BTA19:g.29609605-29609615del), which are both in exon 24 of the *MYH3* gene. The two variants lie 18 bp apart from each other and may result from the same mutation event. These variants were tested by Sanger sequencing on the 25 CP-affected animals and on the parents and relatives, for which DNA was available. All the affected animals were homozygous for the alternate alleles, all the parents were heterozygous for the alternate alleles, and none of the relatives was homozygous for the alternate alleles, which is consistent with the recessive mode of inheritance of the disease.

In total, 834,792 animals from 14 French cattle breeds were genotyped with the EuroGMD SNP chip. The alternate alleles were absent from all the breeds except the Limousine breed. In the Limousine breed, the alternate allele frequency was estimated to be 2.4% (see Additional file 4: Table S3).

### The two mutations result in loss-of-function of MYH3

Bovine *MYH3* is a large gene encompassing 43 exons, which encodes the 1940 amino-acids of the embryonic isoform of the myosin heavy chain protein (MYH3 or MyHC emb). The two identified mutations result respectively in an amino acid substitution (p.Ile955Thr) that is predicted to have a deleterious effect, based on the SIFT and PROVEAN prediction software (PROVEAN score − 4.076), and in a frameshift producing a premature stop codon (p.Leu958_Leu962delinsThrGlyGlnGlyTer) in exon 24, truncating the MYH3 protein of most of its tail domain (Fig. 3c, d). Consequently, each of these two mutations is predicted to be highly deleterious.

**Fig. 4:**
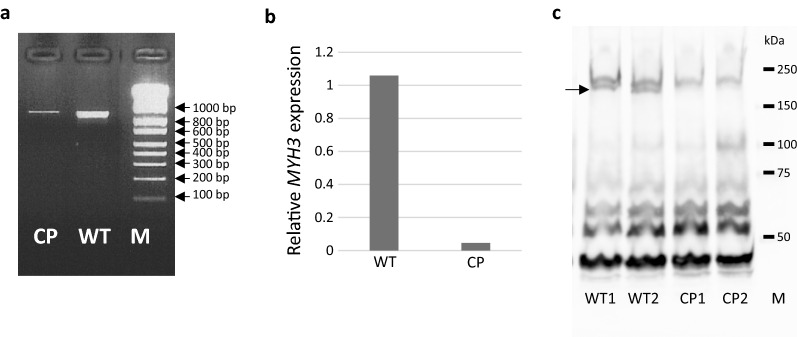
Functional consequences of mutations in the *MYH3* gene. **a** Result of the RT-PCR from CP and WT oculomotor muscles with primers MYH3ex21.1 and MYH3ex27.2 spanning exons 21 to 27 demonstrates that *MYH3* mRNA expression is reduced in affected animals, but no alternative transcript resulting from the deletion could be identified. *M* marker, *bp* base pairs, *WT* wild-type, *CP* cleft palate. **b** Relative expression of *MYH3* from CP and WT oculomotor muscles. The RT-qPCR of *MYH3* exon 23 confirms the mRNA decay. The mRNA levels were normalized with the *RPL13A* and *SDHA* genes. *WT* wild-type, *CP* cleft palate. **c** Proteins were extracted from CP and WT oculomotor muscles. Samples were analysed by immunoblotting with an antibody against the MYH3 protein. At the protein level, the mutation led to a functional knockout as no MYH3 protein was identified by western blot in CP cases. *M* marker, *kDa* kilo Dalton, *WT* wild-type, *CP* cleft palate

MYH3 belongs to the family of myosin heavy chain proteins, which ensure contractile function in the muscles. MYH3 is more specifically expressed in skeletal muscles during embryogenesis in mammals including mice and cattle [46, 47]. After birth, its expression is restricted to the extraocular muscles in cattle [47]. Thus, in order to study the effect of mutations in the *MYH3* gene, we collected post-mortem extraocular muscles from WT and CP calves. *MYH3* mRNA expression was analysed by RT-PCR. Affected calves showed a reduced mRNA level of expression (Fig. 4a). RT-PCR products were sequenced and transcripts from the CP-affected animals only exhibited the two mutations (data not shown). The drastic decrease in *MYH3* mRNA expression in the extraocular muscles from the CP cases was confirmed by RT-qPCR (Fig. 4b). It suggests a nonsense-mediated mRNA decay mechanism that was expected with the presence of the premature termination codon [48].

Western blot of protein extracts from WT and CP extraocular muscles revealed the presence of a ~ 220 kDa band in WT tissues, corresponding to the WT MYH3 protein (molecular weight: 223 kDa), and the absence of detectable truncated MYH3 protein in CP tissues, as expected based on the mRNA expression level (Fig. 4c). The other bands on the western blot probably originated either from a lack of specificity of the primary antibody, which is not specific to cattle, or from detection by the secondary antibody, since these additional proteins are present in similar amounts in WT and CP samples. Indirectly, they confirm the equal loading of protein amounts between the different samples.

## Discussion

In this study, we report a novel aetiology of CP in Limousine cattle, with a cleft of the soft and hard palates (Veau II) in all reported calves. The majority of the cases exhibited non-syndromic CP. For the few syndromic CP cases, clinical signs were variable among the individuals and concerned mainly the musculo-skeletal system, as reported when CP is associated with arthrogryposis. Homozygosity mapping combined with high-throughput sequencing led to the identification of two closely-linked mutations in exon 24 of the *MYH3* gene. All the CP animals were homozygous for these two mutations. Among the genotyped animals, no recombinant individual was detected. Therefore, it is likely that these two mutations resulted from a single event. The substitution is predicted to be deleterious, and the frameshift deletion leads to reduced mRNA levels, which likely result from nonsense-mediated mRNA decay. The resulting protein is predicted to be substantially truncated, and was not detected by western blot. Thus, the mutations induce a loss-of-function of MYH3.

MYH3 is expressed in skeletal muscles, more specifically during development [46] and is not reported to be expressed in the palate. However, palate and muscle development are closely interconnected (reviewed in [49]). Palatal buds elevate along the developing tongue, and first muscular movements, which lower the tongue, are necessary for the horizontal growth of the buds above the tongue. As shown with the study of several mouse mutants and human diseases, malformation (Hoxa2 mutant, [50]), dysfunction or misplacement (csp1^−/−^ mice, [51]) of the tongue can indeed disturb palate development and induce a cleft palate. Moreover, a paracrine influence of the tongue muscles on surrounding tissues such as palatal buds has also been suggested [52]. Consequently, loss-of-function of MYH3 can have an impact on palate development in cattle and the described mutations are most likely causing the bovine CP phenotype.

In humans, dominant non-synonymous *MYH3* mutations cause distal arthrogryposis such as in the Freeman-Sheldon (FSS) and Sheldon-Hall (SHS) syndromes, which are characterized by multiple facial and limb congenital contractures [53]. In these syndromes, CP has not been described. These mutations mainly affect the head domain of the myosin, and disrupt its ATPase activity. Drosophila transgenic models were generated for three of the most frequent FSS mutations, and their study showed that such mutations lead to structural muscular abnormalities, which impair muscle function [54]. Other dominant or compound heterozygous mutations in humans are associated with spondylocarpotarsal synostosis syndrome (SCT), a rare disease characterized by vertebral, carpal and tarsal fusions, short stature and scoliosis, and incomplete penetrant CP [55–57]. Notably, in contrast to our findings in cattle, no human was found to be homozygous or compound heterozygous for a frameshift-truncating variant of *MYH3*.

Agarwal et al. [58] performed knock-out of the *Myh3* gene in mice by deleting exons 3 to 7, which resulted in embryonic loss of about a half of the mice pups that were homozygous for the deletion. Viable homozygous *Myh3*^*Δ/Δ*^ animals developed scoliosis, reminiscent of SCT, but no CP was reported. *Myh3*^*Δ/Δ*^ mice exhibited neonatal and postnatal alterations of the muscle development that resulted from the misregulation of genes involved in muscular differentiation [58], thus confirming the role of MYH3 in myofiber-type specification [59]. The absence of reports of CP in their experiment could suggest either that the mutation in *Myh3* is necessary but not sufficient to induce the phenotype in the absence of other modifier genes at least in mice, or that the authors did not notice this alteration that could explain the observed loss of homozygous mice. However, the difference in phenotype could also be due to species differences in terms of MYH3 expression. Experiments are underway to conclude between these options.

Thus, compared to the existing mutations in the human and murine *MYH3* genes, the recessive CP cases observed in Limousine cattle are particularly original, because in this species, CP is the most preponderant clinical sign.

## Conclusions

In conclusion, we identified likely causal mutations for recessive cleft palate in Limousine cattle. This result has already been exploited and has permitted to manage the disease in the Limousine breed, by including the mutations on the SNP chips used by breeders. Cattle is the first species where loss-of-function of MYH3 preferentially leads to a defect of the palatogenesis rather than to arthrogryposis or to skeletal defects. Additional studies are necessary to further understand the specific role of MYH3 in craniofacial development and to more precisely understand the interactions between the tongue and palatal buds during palatogenesis.

## Supplementary Information

**Additional file 1: Table S1.** List of the disorders in animals (except man, mouse, rat and zebrafish) which include “cleft palate” as a clinical sign. All the data originate from OMIA (Online Mendelian Inheritance in Animals, www.omia.org). All the cattle disorders are written in bold. AR: autosomal recessive; AD: autosomal dominant; UK: unknown.

**Additional file 2: Table S2.** Characteristics of the 26 Limousine CP-affected animals described in this study.

**Additional file 3: Figure S1.** Results of the homozygosity mapping on all the bovine chromosomes. LRT: likelihood-ratio test; BTA: *Bos taurus* chromosome.

**Additional file 4: Table S3.** Frequency analysis of the *MYH3* mutations in 834,792 animals from 14 commercial breeds based on genotyping with EuroG10k v8 and EuroGMD SNP chips. The two mutations are totally linked, thus the frequency is the same for both mutations. freq_WTall: frequency of the WT allele; freq_MUTall: frequency of the mutated allele.

## Data Availability

Whole-genome sequence data for the two affected animals are deposited in the European Nucleotide Archive under the study accession number PRJEB51730.
