# Supplementary figures and images for: Mutation of the MYH3 gene causes recessive cleft palate in Limousine cattle
Source: Genet Sel Evol. 2022 Oct 29;54:71. doi: 10.1186/s12711-022-00762-2 (PMC9617432; doi:10.1186/s12711-022-00762-2)

## Slide 1
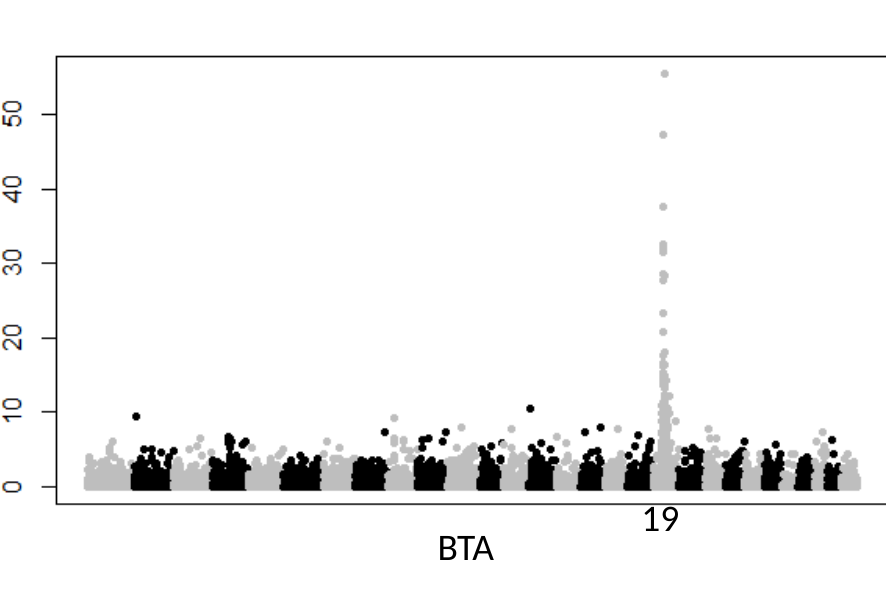

19
BTA

Supplement: Supplementary file 3 — Additional file 3: Figure S1. Results of the homozygosity mapping on all the bovine chromosomes. LRT: likelihood-ratio test; BTA: Bos taurus chromosome. [file 12711_2022_762_MOESM3_ESM.pptx]
